# Genome analysis of *Erwinia persicina* reveals implications for soft rot pathogenicity in plants

**DOI:** 10.3389/fmicb.2022.1001139

**Published:** 2022-10-28

**Authors:** Chloe Wasendorf, Stephan Schmitz-Esser, Carter J. Eischeid, Martin J. Leyhe, Erika N. Nelson, Faith M. Rahic-Seggerman, Kasey E. Sullivan, Nick T. Peters

**Affiliations:** ^1^Department of Plant Pathology and Microbiology, Iowa State University, Ames, IA, United States; ^2^Biology Division, Alfred University, Alfred, NY, United States; ^3^Department of Animal Science, Iowa State University, Ames, IA, United States; ^4^Interdepartmental Microbiology Graduate Program, Iowa State University, Ames, IA, United States; ^5^Department of Chemistry, Iowa State University, Ames, IA, United States; ^6^Undergraduate Microbiology Program, Iowa State University, Ames, IA, United States

**Keywords:** soft rot, *Erwinia persicina*, sequencing, plant pathogen, genome

## Abstract

Soft rot disease causes devastating losses to crop plants all over the world, with up to 90% loss in tropical climates. To better understand this economically important disease, we isolated four soft rot-causing *Erwinia persicina* strains from rotted vegetables. Notably, *E. persicina* has only recently been identified as a soft rot pathogen and a comprehensive genomic analysis and comparison has yet to be conducted. Here, we provide the first genomic analysis of *E. persicina*, compared to *Pectobacterium carotovorum*, *P. carotovorum*, and associated *Erwinia* plant pathogens. We found that *E. persicina* shares common genomic features with other *Erwinia* species and *P. carotovorum*, while having its own unique characteristics as well. The *E. persicina* strains examined here lack Type II and Type III secretion systems, commonly used to secrete pectolytic enzymes and evade the host immune response, respectively. *E. persicina* contains fewer putative pectolytic enzymes than *P. carotovorum* and lacks the Out cluster of the Type II secretion system while harboring a siderophore that causes a unique pink pigmentation during soft rot infections. Interestingly, a putative phenolic acid decarboxylase is present in the *E. persicina* strains and some soft rot pathogens, but absent in other *Erwinia* species, thus potentially providing an important factor for soft rot. All four *E. persicina* isolates obtained here and many other *E. persicina* genomes contain plasmids larger than 100 kbp that encode proteins likely important for adaptation to plant hosts. This research provides new insights into the possible mechanisms of soft rot disease by *E. persicina* and potential targets for diagnostic tools and control measures.

## Introduction

Soft rot diseases of plants cause crop loss all over the world. In temperate climates, soft rot disease can cause 15–30% of crop loss, while in tropical climates, it can cause up to 90% of crop loss (Van Gijsegem et al., 2021). These losses contribute significantly to decreased resources for subsistence farmers, decreasing the ability to feed the growing global population (United Nations, 2013). For example, economic losses due to soft rot diseases of potatoes in Europe are estimated to be 46 million euros annually (Dupuis et al., 2021). However, potatoes are not the only crop effected. Soft rot bacteria can cause disease on a wide range of produce, from Solanaceae to cucurbits to Apiaceae (Toth et al., 2021).

Soft rot disease initially presents as watersoaked lesions on harvested crops. The mushy lesions that develop are due to maceration of plant tissue by pectolytic enzymes secreted by soft rot agents (Toth et al., 2021). Pectolytic enzymes degrade the pectin between plant cell walls in the middle lamella, thereby damaging cellular integrity, leading to cell death. Also, proteases and cellulases often play an assessory role to the pectolytic enzymes, furthering tissue destruction. Symptoms typically appear post-harvest, while crops are in storage, but may also appear while crops are still growing in the field. Once symptoms become visible, the crop is no longer useful as a food source. Thus, there is no treatment for soft rot, only preventative measures are currently available.

The most common bacterial soft rot agents are *Pectobacterium* and *Dickeya* species, which until the late 1990s were part of the *Erwinia* genus (Hauben et al., 1998). Genetic comparisons of the 16S rRNA genes of 29 *Erwinia, Pantoea,* and *Enterobacter* species found that the organisms that caused soft rot should be assigned their own genera, *Pectobacterium* and *Dickeya* (Hauben et al., 1998; Samson et al., 2005). Recently, another species within the *Erwinia* genus, *Erwinia persicina*, has been identified as a soft rot agent (Gálvez et al., 2015; Cho et al., 2019; Nechwatal and Theil, 2019; Canik Orel, 2020). *Erwinia persicina* causes pink-pigmented soft rot on a small range of plant hosts, including: garlic, onions, lettuce, mushrooms, barley and parsley root (Gálvez et al., 2015; Cho et al., 2019; Nechwatal and Theil, 2019; Yan et al., 2019; Canik Orel, 2020; Kawaguchi et al., 2021). Prior to the identification as a soft rot agent, *E. persicina* was classified as an epiphyte of cucumbers, tomatoes, and bananas; there has been one report of isolation from the urinary tract of a human (Hao et al., 1990; O’Hara et al., 1998). It was also identified as the causative agent of necrotic leaf spots on legumes and leaf wilting of alfalfa (González et al., 2007; Zhang and Nan, 2012; Zhang and Nan, 2014). However, no comprehensive genomic analysis of *E. persicina* has been previously published.

The *Erwinia* genus contains other plant pathogens that are not soft rot agents. Most notable are *Erwinia amylovora, Erwinia tracheiphila,* and *Erwinia pyrifoliae*. Both *E. amylovora* and *E. pyrifoliae* cause necrotic diseases of woody trees in the *Rosaceae* family, while *E. tracheiphila* causes bacterial wilt of cucurbits (Rhim et al., 1999; Zhao and Qi, 2011; Rojas et al., 2013). Bacterial wilt is characterized by occlusion of the xylem with bacteria and their associated polysaccharide secretions, thereby impairing water transport from the roots to the shoots (Sarkar and Chaudhuri, 2016). Necrotic diseases involve the death of plant tissues, like leaves, stems, or branches, due to the secretion of effector molecules and exopolysaccharides. Soft rot diseases are similar to necrotic diseases in that both result in the destruction of plant tissues, albeit on a completely different scale with specific enzymes being employed in each disease. The genomes of *Erwinia* pathogens, including *E. amylovora, E. pyrifoliae, E. tracheiphila,* and non-pathogen *Erwinia tasmaniensis*, have all been well characterized and comparative analyzes have determined both species-specific and shared virulence factors (Palacio-Bielsa et al., 2012).

The goal of this work is to provide the first comprehensive analysis of an *E. persicina* genome and compare it to other *Erwinia* and soft rot-causing species, thereby providing avenues for future research to test the functionality of putative genes and molecular pathways.

## Materials and methods

### Isolation of soft rot bacteria

Bacteria were isolated from green onion and asparagus showing signs of soft rot disease. The samples were collected from store-discarded vegetables (Ames, IA) that were allowed to further rot in the laboratory at room temperature under humid conditions. Cut carrot slices were used to distinguish soft rot causing bacteria from other saprophytic bacteria isolated from diseased vegetables. Samples of rotten tissues were inoculated onto 70% ethanol sterilized carrot slices and incubated in a moist chamber at 30°C for 48 h. Samples from carrot slices that showed signs of soft rot were directly inoculated onto Luria-Bertani Broth (LB) plates and incubated at 30°C overnight to acquire isolated colonies. Each colony was then inoculated onto a fresh, sterilized carrot slice and incubated in a moist chamber at 30°C for 48 h to test soft rot capabilities. Isolates that showed soft rot capacity were given an isolate designation and preserved for further experiments.

### Soft rot host range testing of isolates

Potatoes, radishes, carrots, onions (both white and yellow), and garlic were chosen for host range determination experiments. These vegetables were selected as they are commonly used in soft rot host range assays and potatoes are considered a staple food product across the world (Savary et al., 2019). Potatoes and radishes were kept whole and, if present, the stem and roots of the radishes were removed. Onions were sliced into 1-inch portions and separated into layers. A cavity was then formed by gouging the surface of the vegetables with a 10 μl pipette tip before inoculation of 10 μl of stationary phase bacterial cultures of each of the four isolates, separately (all bacterial cultures grew to 10^9^ CFU/ml in LB after overnight incubation at 30°C). Carrots were prepared as described above. All vegetables were washed with 50% ethanol and distilled water before inoculation. All assays were conducted at 30°C for 48–72 h in a moist chamber before images were taken and results recorded. Bacterial cultures were grown overnight in LB broth at 30°C at 200 rpm. Prior to inoculation, cells were washed and resuspended in a phage buffer solution (10 mM Tris (pH 7.5), 10 mM MgSO_4_, 68 mM NaCl, 1 mM CaCl_2_, distilled water, then filter sterilized) to remove any growth media.

### DNA extraction and genome sequencing

Pure cultures of each isolate were grown overnight in LB broth at 30°C while shaking at 200 rpm. The Nanobind CBB Big DNA Kit (Circulomics, Baltimore, MD, United States) was then used to extract high molecular weight DNA following the instructions of the manufacturer. Sequencing was conducted using Illumina MiSeq 250 bp read length paired-end sequencing at the ISU DNA Facility. Library preparation was performed using the NEBNext Ultra II FS kit with standard parameters. FastQC v0.11.9 was used to assess the quality of reads (note: default parameters were used for all software unless specified otherwise) (Andrews, 2010). Bases below a quality score of 20 were trimmed and adapter sequences were removed with BBDuk v37.36 using the following options: “ref = adapters.fasta ktrim = r ordered k = 23 hdist = 1 mink = 11 tpe tbo qtrim = w trimq = 20 minlen = 75” (Bushnell, 2014). Only reads greater than 75 bp after trimming were used to generate initial genome assemblies with SPAdes v3.14.1 using the “--careful” option (Bankevich et al., 2012). Based on average nucleotide identities (ANI) between the isolates, calculated using JSpeciesWS (Richter et al., 2016), strain SR15 was chosen for additional sequencing with Oxford Nanopore GridION technology to obtain a closed genome, using the same DNA samples used in the Illumina MiSeq sequencing run. Library preparation for Oxford Nanopore sequencing was performed using the SQK-LSK109 kit with barcoding kit EXP-NBD104 with standard parameters. The Illumina MiSeq and Nanopore reads were used to generate hybrid genome assemblies using Unicycler v0.4.8 (Wick et al., 2017). Annotation of assembled genomes was performed through the Patric database and the NCBI PGAP (Tatusova et al., 2016; Davis et al., 2019).

### Isolate genus and species classification

To determine the genus and species of the isolates, average nucleotide identities (ANIs) were calculated and a tetra correlation search (TCS) was conducted using JSpeciesWS (Richter et al., 2016). Reference sequences used were derived from GenomesDB (which is included in the Jspecies Webserver) (Richter et al., 2008) and NCBI to compare the *E. persicina* SR13-16 genomes to other *Erwinia* species and common soft rot agents’ genomes. The heatmap showing the ANI results was generated using JcolorGrid (Joachimiak et al., 2006). JSpeciesWS also provided a TCS that compiled the most similar genomes, from Genomes DB, to *E. persicina* SR15 and calculated correlation values to quantify the similarity (Richter and Rosselló-Móra, 2009).

16S rRNA genes from *E. persicina* SR13-16 isolates, *Erwinia* species type strains, and common soft rot bacterial pathogens were used to build the phylogenetic tree. Sequences of the 16S rRNA genes were collected from NCBI. Evolutionary analyzes were conducted in MEGA11 (Tamura et al., 2021). The evolutionary history was inferred by using the Maximum Likelihood method and Tamura-Nei model (Tamura and Nei, 1993). The percentage of trees, from 500 rounds, in which the associated taxa clustered together is shown below the branches. Initial trees for the heuristic search were obtained automatically by applying Neighbor-Join and BioNJ algorithms to a matrix of pairwise distances estimated using the Tamura-Nei model, and then selecting the topology with superior log likelihood value. This analysis involved 44 16S rRNA gene sequences. There were a total of 1,573 positions in the final dataset.

### Specific comparisons of selected candidate proteins important for causing disease in plants

Proteins important for causing disease and colonizing the plant host for non-*Erwinia* soft rot pathogens and other *Erwinia* pathogens were selected for comparison to *E. persicina* isolates (Smits et al., 2010; Li et al., 2018). The organisms examined in the protein comparisons are described in Table 1. The query protein sequences were selected from a variety of organisms including: *Pectobacterium carotovorum* subsp. *odiferum* BC S7 (BCS7 locus tags), *P. carotovorum* SCC1 (SCC1 locus tags), *Dickeya dadantii* 3937 (Dda3937 locus tags), *E. amylovora* CFBP1430 (EAMY locus tags), *P. carotovorum* SCRI193 (CAA locus tags), *Pectobacterium atrosepticum* SCRI1043 (ECA locus tags), *Erwinia rhapontici* P45 (AMB locus tags), *E. persicina* SR15 (NOG67_11500) (Reeves et al., 1993; Bell et al., 2004; Smits et al., 2010; Glasner et al., 2011; Born et al., 2016; Niemi et al., 2017; Li et al., 2018). Multiple organisms were used as sources for protein sequences to encompass the important proteins needed to cause disease and survive in the plant environment for non-*Erwinia* and *Erwinia* soft rot pathogens, other *Erwinia* phytopathogens, and non-pathogen, plant-associated *Erwinia*.

**Table 1:** Bacterial genomes used in protein comparisons.

Organism	Reason	Disease phenotype	Accession numbers	References
*Erwinia amylovora* CFB1430	Phytopathogen	Necrotic disease of pome fruits; fire blight	FN434113-FN434114	Smits et al. (2010)
*Erwinia persicina* B64	*Erwinia persicina*	Pink soft rot	CP022725-CP022727	Cho et al. (2019)
*Erwinia persicina* NBRC 102418	*Erwinia persicina*	Pink soft rot	BCTN00000000	Hao et al. (1990)
*Erwinia persicina* SR13/14	Subject	Pink soft rot	JANFMX000000000, JANFMY000000000	This study
*Erwinia persicina* SR15/16	Subject	Pink soft rot	CP101613-CP101614, JANFMZ000000000	This study
*Erwinia rhapontici* MAFF 311153	Phytopathogen	Pink seed and crown rot	AP024329-AP024330	Morohoshi et al. (2021)
*Erwinia tasmaniensis* Et1/99	Non-pathogen	Non-pathogen	CU468135, CU468128, CU468130, CU468131, CU468132, CU468133	Kube et al. (2008)
*Pectobacterium carotovorum* WPP14	Phytopathogen	Soft rot	CP051652	Glasner et al. (2008)

## Results and discussion

This study has provided the first detailed insights in the genomic makeup of *E. persicina* as a soft rot agent, as well as genomic comparisons with a common soft rot agent, *P. carotovorum*, and other *Erwinia* pathogens that do not cause soft rot and non-pathogens. As of yet, there have been no publications investigating the genome of *E. persicina* other than to briefly describe the genome sequence of *E. persicina* strain B64 (Cho et al., 2019). Our current work thus significantly expands the knowledge of the *E. persicina* genome by discussing the absence of the Out cluster of the T2SS, which is used by well-characterized soft rot pathogens to secrete pectolytic enzymes needed for disease, and the presence of a phenolic acid decarboxylase, a possible adaptation to survive within the plant host environment that is not present in other *Erwinia* plant pathogens. The comparisons with another soft rot pathogen and *Erwinia* pathogens that do not cause soft rot provides a better understanding of where these isolates fit among plant pathogens.

### Isolates SR13-16 are *Erwinia persicina* strains

**Figure 1:**
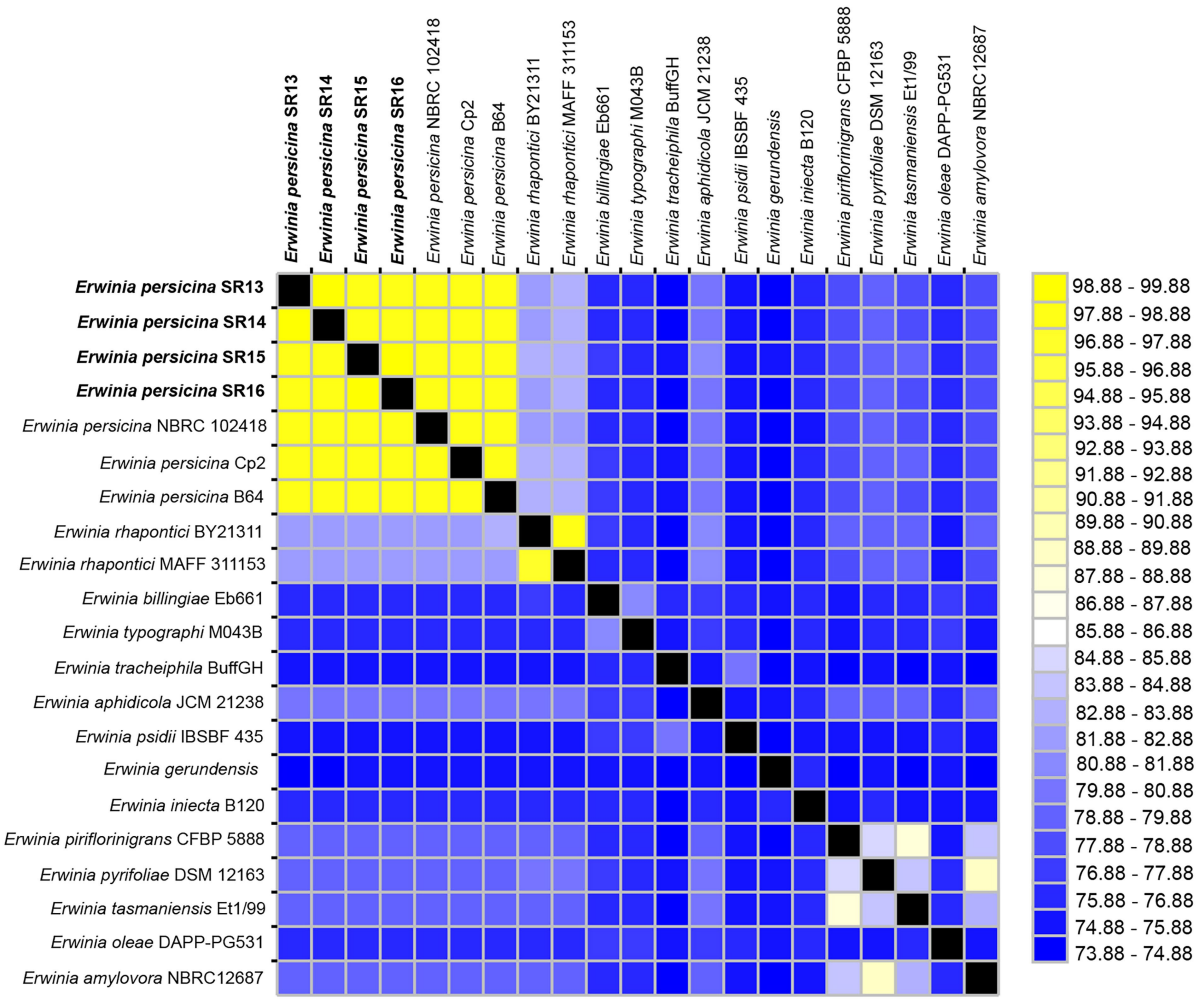
Average nucleotide identities (ANI) between *Erwinia persicina* SR13-16 and other *Erwinia* species. ANI analysis was conducted using the JSpeciesWS webserver with sequences from GenomesDB and NCBI. The heatmap was generated using JColorGrid. ANI values range from 73–100% (blue to yellow). *E. persicina* SR13-16 are most similar to each other and to other *E. persicina* strains.

**Figure 2:**
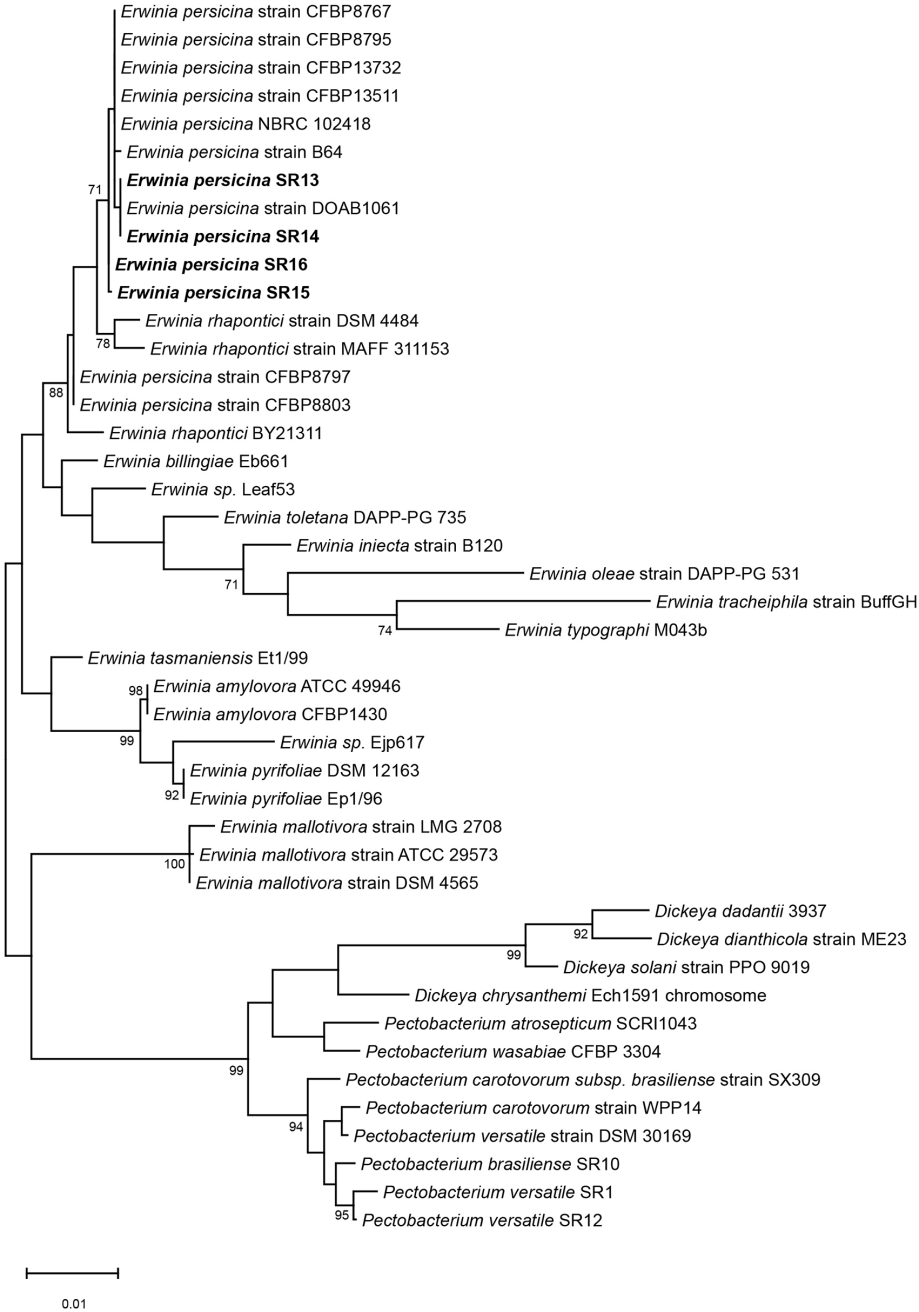
Phylogenetic tree using 16S rRNA genes of *E. persicina* SR13-16, other *Erwinia* species and common soft rot agents. The 16S rRNA genes of *E. persicina* SR13-16 isolates (in bold), various *Erwinia, Dickeya,* and *Pectobacterium* spp. strains were used to build the tree. Sequences of the 16S rRNA genes were collected from NCBI. The evolutionary history was inferred by using the Maximum Likelihood method and Tamura-Nei model. The tree with the highest log likelihood (−5331.53) is shown. The percentage of trees in which the associated taxa clustered together is shown below the branches. Initial tree(s) for the heuristic search were obtained automatically by applying Neighbor-Join and BioNJ algorithms to a matrix of pairwise distances estimated using the Tamura-Nei model, and then selecting the topology with superior log likelihood value. The tree is drawn to scale, with branch lengths measured in the number of substitutions per site. This analysis involved 44 nucleotide sequences. There were a total of 1,573 positions in the final dataset. Evolutionary analyzes were conducted in MEGA11.

Sampling rotten vegetables yielded several species of soft rot causing bacteria. Four *E. persicina* isolates (SR13-16) were selected for further investigation due to their relative uniqueness in causing soft rot and the lack of detailed genomic analyzes conducted on this species. PCR amplification, Sanger sequencing, and BLAST analysis of their 16S rRNA genes identified the isolates as *E. persicina* strains and further Illumina sequencing to obtain their draft genomes confirmed their classification (Table 2). ANIs within the group were over 99.9% identical to each other (Figure 1). Due to the high similarities within the *E. persicina* genome group, one isolate, SR15, was chosen for further sequencing with Oxford Nanopore GridION to obtain a closed genome. All four genomes ranged in size from 4.81 to 4.89 Mbp and contained 2–55 contigs (Table 3). All isolates contain a chromosome of approximately 4.7 Mbp and a plasmid of 148 or 165 kbp (Table 3). The ANI analysis showed that there were two subgroups within the four isolates. Strains SR13 and 14 were virtually identical (100% ANI and over 99.8% coverage), as were strains SR15 and 16 (100% ANI and over 99.6% coverage) (Figure 1). The ANI values were above the threshold of species demarcations (ANI > 95%, 16S rRNA gene > 99%) (Figure 1; Chun and Rainey, 2014; Varghese et al., 2015). The TCS indicated the isolates were *E. persicina* strains with correlation values over 0.99970 when comparing to *E. persicina* NBRC 102418 indicating it is likely of the same species (TCS values > 0.99) (Richter and Rosselló-Móra, 2009). The phylogenetic tree based on 16S rRNA gene sequences shows a distinct grouping of isolates SR13-16 with other *E. persicina* strains with high bootstrap support and distance from the outgroup of *Pectobacterium* and *Dickeya* species (Figure 2). The *Erwinia* species that the *E. persicina* isolates cluster closest with other than *E. persicina* strains was *E. rhapontici* strain DSM 4484 which was also reflected by the ANI analyzes (Figure 1). Together, this information confirms that isolates SR13-16 are indeed *E. persicina* strains.

**Table 2:** Genome sequencing raw read data.

*E. persicina* isolate	No. of Illumina reads	Illumina sequenced (Mbp)	No. of nanopore reads	Nanopore sequenced (Gbp)	Nanopore N50 (Kbp)
SR13	1,112,824	278.2			
SR14	1,137,738	284.4			
SR15	1,320,914	330.2	98,958	1.355	33.31
SR16	1,563,720	390.9			

**Table 3:** *Erwinia persicina* soft rot isolate genome characteristics.

*E. persicina* isolate	Sequencing platform	Chromosome size (Mbp)	Plasmid size (Kbp)	No. of contigs	GC %	Isolation source	Accession numbers
SR13	Illumina	4.66	148	30	55.6	Asparagus	JANFMX000000000
SR14	Illumina	4.65	148	30	55.5	Asparagus	JANFMY000000000
SR15	Illumina, Nanopore	4.73	156	2^a^	55.4	Green onion	CP101613-CP101614
SR16	Illumina	4.71	156	55	55.4	Green onion	JANFMZ000000000

a One contig is the closed genome and the other is the plasmid.

### *Erwinia persicina* SR13-16 cause soft rot on various vegetables

**Figure 3:**
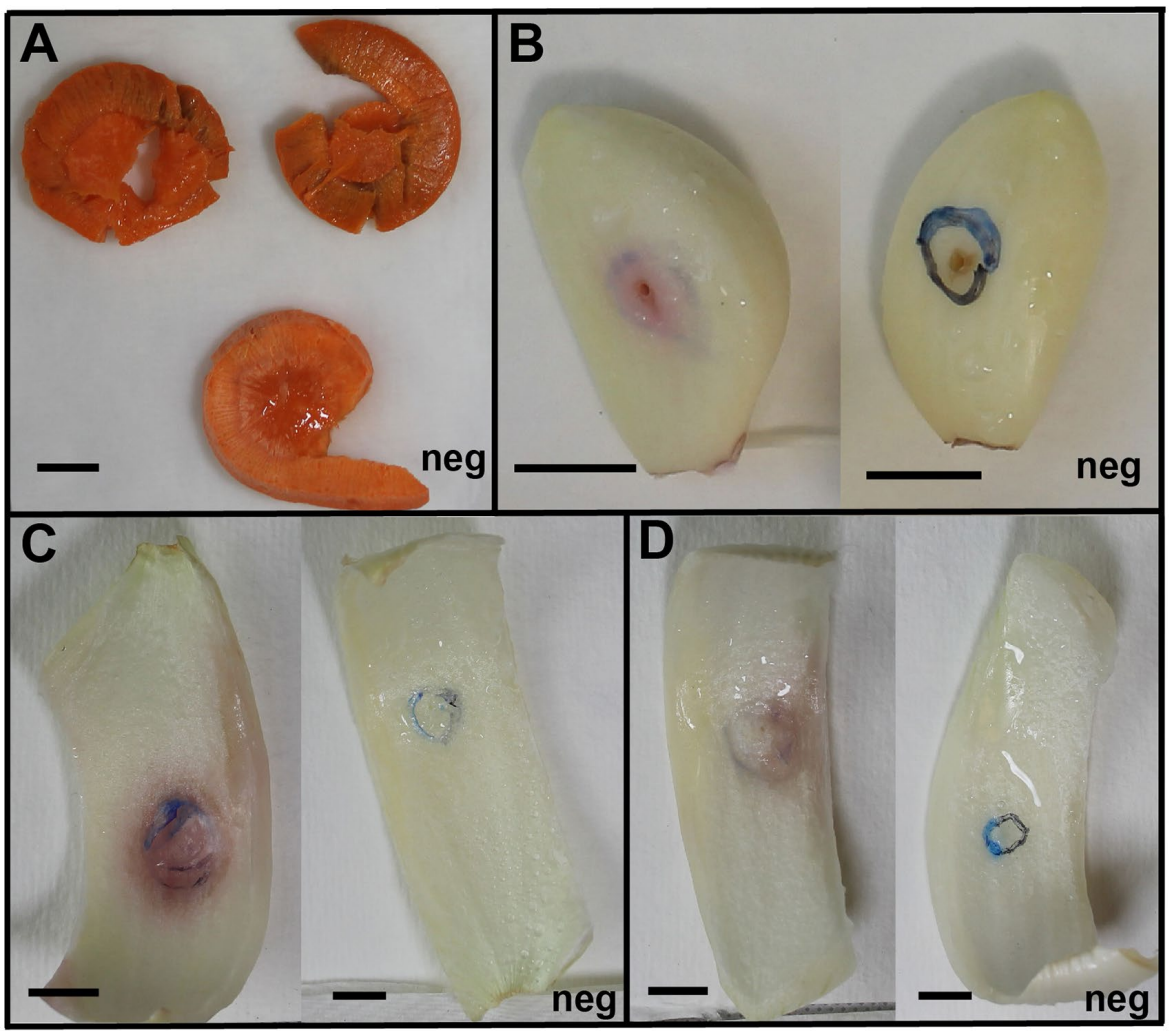
*Erwinia persicina* SR13-16 cause soft rot on carrots, garlic, white and yellow onions. Examples of soft rot caused by *E. persicina* SR13-16 isolates. All vegetables were incubated at 30°C and pictures were taken after 48 h for the yellow onion **(C)** and white onion **(D)**, 72 h for the carrots **(A)**, and 96 h for the garlic **(B)**. Negative controls, inoculated with sterile buffer solution, are marked with “Neg” in each panel. Scale bars equal 1 cm. A pink pigmentation accompanies soft rot symptoms on garlic and both onion varieties **(C,D)**.

**Figure 4:**
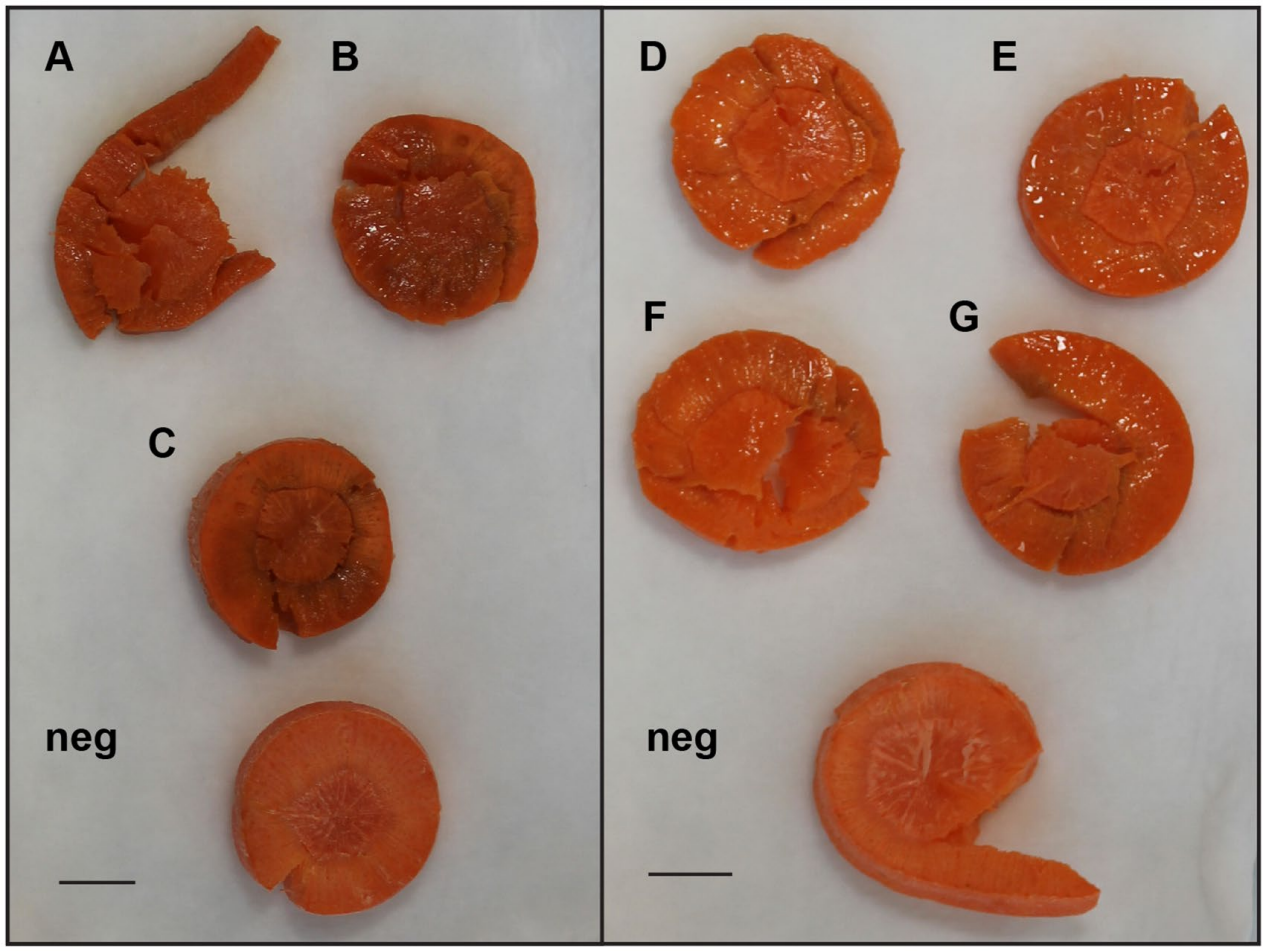
Examples of differences in soft rot caused by select *E. persicina* and *Pectobacterium* isolates after 48 h. To visualize differences in virulence, sterilized carrot slices were inoculated with 10 μl of overnight cultures of soft rot causing isolates that were washed and resuspended in a buffer solution and incubated at 30°C for 48 h. Isolates used were *Pectobacterium versatile* SR1 **(A)**, *Pectobacterium brasiliense* SR10 **(B)**, *P. versatile* SR12 **(C)**, and *E. persicina* SR13-16 [**(D–G)**, respectively]. Negative controls (Neg) were inoculated with sterile buffer solution. Scale bar equals 1 cm. *E. persicina* isolates are still in the first stages of causing soft rot symptoms (water soaking), while the *Pectobacterium* isolates have already progressed to the latter stages of the disease (darkened spots of tissue maceration).

Currently, *E. persicina* has a small known host range and characteristically causes pink soft rot on garlic, onion, lettuce, and parsley root (Gálvez et al., 2015; Cho et al., 2019; Nechwatal and Theil, 2019; Canik Orel, 2020). Isolates SR13-16 caused rot symptoms on carrots, garlic cloves, and white and yellow onions (Figure 3). All *E. persicina* isolates produced similar soft rot symptoms on each vegetable. When inoculated onto carrots, the isolates were slower to induce soft rot and were less consistent than positive controls using *Pectobacterium brasiliense* strain SR10 and *Pectobacterium versatile* strains SR1 and SR12 (Wasendorf et al., 2022), and no pink pigmentation was observed (Figure 4). At 48 h after inoculation, carrots inoculated with the *E. persicina* isolates were still in the first stages of soft rot symptoms, often called water soaking due to the initial release of cytoplasmic contents from lysed plant cells, while carrots inoculated with the *Pectobacterium* controls had darkened spots of macerated tissue, which are more advanced symptoms of the disease. *E. persicina* isolates did produce a pink pigment while causing soft rot on garlic and onion, which aligns with previous research (Gálvez et al., 2015; Cho et al., 2019; Nechwatal and Theil, 2019; Canik Orel, 2020). When a pink color was observed on the onions, it was accompanied by signs of tissue maceration. *E. persicina* was found to cause rot on potato tuber slices in one previously published study, but the *E. persicina* strains examined here did not cause soft rot symptoms on whole potatoes or whole radishes and were not examined on potato slices (Nechwatal and Theil, 2019). It has been reported that during *in vitro* soft rot pathogenicity tests on parsley root, using pure isolates of *E. persicina,* the pathogen produced fewer and less severe soft rot symptoms (Nechwatal and Theil, 2019). However, the pink pigmentation appeared every time there was soft rot, as well as when there were no symptoms, implying that *E. persicina* was present, but not always causing soft rot. *E. persicina* also seems to cause soft rot symptoms slower than *Pectobacterium* species but was isolated multiple times from different rotten vegetables. It may be that *E. persicina* is not the main soft rot agent in the field, but that does not mean that it is not an important pathogen to consider as it was isolated multiple times from different rotten vegetables. There is clearly much more to learn about the community dynamics of soft rot causing bacteria.

### *Erwinia persicina* strains have similar genetic traits as common soft rot agents and other *Erwinia* pathogens

#### Pectolytic genes

To better understand the mechanisms involved in causing soft rot and survival in the plant environment for the *E. persicina* strains, BLAST analyzes were conducted using proteins that are important to both of those functions. Firstly, the genomic abundance of putative pectolytic genes was determined. *E. persicina* strains and other *Erwinia* species examined in this study have a fewer number of putative pectolytic enzyme genes (4–9) than *P. carotovorum*, a common soft rot agent (19) (Table 4). When comparing within the *E. persicina* strains in this study, the majority of pectolytic enzyme genes were conserved. The largest difference between the strains was *E. persicina* B64, which contains the most pectolytic enzyme genes. Also, enzymes PehK and PehN, both of which are polygalacturonases, are missing from *E. persicina* 102,418 and *E. persicina* SR13/14, respectively, but are present in the other *E. persicina* strains. The lower amount of pectolytic enzyme genes is likely a factor that contributes to *E. persicina* being a less virulent soft rot agent than *Pectobacterium* species. Pectolytic enzymes are important virulence factors for soft rot agents as they break down the pectin in plant cell walls and the middle lamella, ultimately leading to cell lysis, and the characteristic symptoms that follow (Toth et al., 2021). Previous research with *Erwinia chrysanthemi* EC16, now called *Dickeya chrysanthemi* EC16, has shown purified samples of pectolytic enzyme PelA are needed in much higher quantities than PelE to cause the same degree of soft rot symptoms on cucumber slices (Tamaki et al., 1988). This implies that the type of pectolytic enzyme present is also a factor to how a soft rot organism may cause disease. Interestingly, both enzymes are absent from most of the *E. persicina* strains, with PelA in *E. persicina* B64 being the exception. Future research would benefit from extracting the purified enzymes from *E. persicina* and evaluating their ability to cause disease on various vegetables on their own and combined to further elucidate the mechanism behind soft rot caused by *E. persicina*. Understanding which enzymes are critical for disease would provide important targets for treatment and prevention.

**Table 4:** Pectolytic enzymes among soft rot pathogens.

Enzyme type	Gene	Locus tag^a^	Ea^b^	Ep B64	Ep 102418	Ep 13/14	Ep 15/16	Er	Et	Pc
Pectolytic enzymes			Amino acid identity (%)							
Pectate lyase	*pelA*	BCS7_19260	30	37						98
	*pelB*	SCC1_0380								98
	*pelC*	BCS7_19270		26						99
	*pelE*	Dda3937_03371								37
	*pelX*	BCS7_21380		53	53	53	52	52		98
	*pelW*	BCS7_09865								99
	*pelI*	BCS7_05195								97
	*hrpW*	BCS7_11155	51						55	94
	*pelZ*	BCS7_19275								99
	*pnl*	BCS7_06950	43	63	63	63	63	64		95
Polygalacturonase	*pehA*	BCS7_05200	59	33					62	95
	*pehK*	BCS7_17010	22	27		24	24		23	96
	*pehN*	BCS7_05675	34	31	37		37		34	99
	*pehX*	BCS7_14740							24	97
Pectin acetylesterase	*paeX*	BCS7_09835		58	58	58	58	58		98
	*paeY*	BCS7_15435								93
Pectinesterase	*pemA*	BCS7_15440								86
	*pemB*	SCC1_4277								97
Oligogalacturonase	*ogl*	BCS7_09745		68	68	68	68	69		99

a BCS7 locus tags are from *Pectobacterium* subsp. *odiferum* BC S7; SCC1 locus tags are from *Pectobacterium carotovorum* SCC1; Dda3937 locus tags are from *Dickeya dadantii* 3937; CAA locus tags are from *Pectobacterium carotovorum* SCRI193.

b Organisms are as follows: Ea, *Erwinia amylovora* CFB1430; Ep B64, *Erwinia persicina* B64; Ep 102418, *Erwinia persicina* NBRC 102418; Ep 13/14, *Erwinia persicina* SR13/14; Ep 15/16, *Erwinia persicina* SR15/16; Er*, Erwinia rhapontici* MAFF 311153; Et, *Erwinia tasmaniensis* Et1/99; and Pc, *Pectobacterium carotovorum* WPP14.

#### Secretion systems

Secretion systems type I-VI were investigated with a focus on type II and III, as they harbored important differences among the strains in the study. Each of the five secretion systems compared has a distinct function for plant pathogens. The type I secretion system, responsible for secreting proteases and other small molecules that aid in causing soft rot and other plant diseases (Zhang et al., 1999), is present in *E. amylovora* and the soft rot agent, *P. carotovorum*, but was absent in the *E. persicina* strains, *E. pyrifoliae,* and *E. tasmaniensis.* The type II secretion system is important for secreting the pectolytic enzymes that cause soft rot disease symptoms (Lindeberg et al., 1996; Green and Mecsas, 2016). When the genes that comprise the type II secretion system, a set of 13 *out* genes, were knocked out in *P. carotovorum*, pectolytic enzymes began collecting in the periplasm as they were not being released from the cell (Murata et al., 1990; Reeves et al., 1993). The *E. persicina* strains in this study do not have the cluster of *out* genes in the Type II secretion system like *P. carotovorum* does, but they do have the genes for the Sec secretion pathway, *secABYEG* (76–100% amino acid identities among all strains, Sec data not shown, *out* cluster in Table 5). The Sec secretion pathway allows the enzymes to be released into the periplasm of the bacterial cell, but the *out* cluster is needed for further secretion to the outside of the cell. Future studies will need to be done to determine if another secretion system is compensating for the lack of the type II *out* secretion system in *E. persicina* to allow for the secretion of the pectolytic enzymes needed to cause the soft rot symptoms produced by the pathogen.

**Table 5:** Absence of the *out* cluster from the Type II secretion system in *Erwinia persicina* strains.

Gene	Locus tag^a^	Ea^b^	Ep B64	Ep 102418	Ep 13/14	Ep 15/16	Er	Et	Pc
		Amino acid identity (%)							
*outC*	CAA49644	37						44	99
*outD*	CAA49645	51						53	93
*outE*	CAA49646	58						58	100
*outF*	CAA49647	58						59	100
*outG*	CAA49648	72						76	100
*outH*	CAA49649								99
*outI*	CAA49650	47						47	99
*outJ*	CAA49651	36						34	100
*outK*	CAA49652	29						29	98
*outL*	CAA49653	23						23	99
*outM*	CAA49654								99
*outN*	CAA49655								100
*outO*	CAA49656	50	45	45	45	45		46	99

a CAA locus tags are from *Pectobacterium carotovorum* SCRI193.

b Organisms are as follows: Ea, *Erwinia amylovora* CFB1430; Ep B64, *Erwinia persicina* B64; Ep 102418, *Erwinia persicina* NBRC 102418; Ep 13/14, *Erwinia persicina* SR13/14; Ep 15/16, *Erwinia persicina* SR15/16; Er*, Erwinia rhapontici* MAFF 311153; Et, *Erwinia tasmaniensis* Et1/99; and Pc, *Pectobacterium carotovorum* WPP14.

The type III secretion system (T3SS) is involved in secreting proteins that are involved in modulating or inhibiting the host immune response so the plant pathogen can continue to colonize and cause disease in the plant host (Yuan et al., 2020). A T3SS is present in many other plant pathogens, including *Erwinia* species, and is an important factor in how they bypass the host immune system (Katagiri and Tsuda, 2010). No Type III secretion system (T3SS) was found in any of the *E. persicina* strains used in this study (data not shown). *E. amylovora* had the T3SS with the highest apparent completeness, and *E. pyrifoliae*, *E. tasmaniensis,* and the common soft rot agents only have a partial T3SS. “Apparent completeness” is defined in this manuscript as the set of genes characterized in other publications or used in comparisons in other publications (Smits et al., 2010; Smits and Duffy, 2011). The lack of a T3SS system in *E. persicina* strains is consistent with other soft rot agents (Davidsson et al., 2013). *Pectobacterium* species’ partial T3SS does not seem to contribute to their virulence like it does for most plant pathogens (Kim et al., 2009). T3SS-deficient mutants showed little to no decrease in virulence when compared to wild-type strains (Kim et al., 2009). Soft rot agents do not rely on proteins being secreted by a T3SS to evade the plant immune system. Instead, they do so by remaining in the plant, in “stealth mode,” undetected by the host until a cell density threshold is reached that triggers the “brute force” phase of infection. The “brute force” phase is characterized by production of pectolytic enzymes and maceration of plant tissue that often progresses too quickly for the plant cells to overcome (Gorshkov et al., 2018). The lack of a T3SS is one way that *E. persicina* is more similar to soft rot pathogens than other *Erwinia* pathogens, while the absence of the T2SS demonstrates how it is different from typical soft rot pathogens.

The type VI secretion system core genes are conserved among all strains in this study. The structural proteins, TssA-C, E-H, J-M, VgrR, and Hcp, compose the secretion system apparatus, which is very similar to the sheath, syringe, and baseplate of bacteriophage (Leiman et al., 2009; Chen et al., 2015). The function of the type VI secretion system in soft rot causing bacteria has yet to be fully elucidated as it is a recently discovered secretion system, but it appears to mediate communication between bacterial cells, both antagonistic (transferring a toxin to eliminate competition) and non-antagonistic (transferring a toxin to kill a phage-infected neighbor cell) (Hood et al., 2010; Russell et al., 2014). It was first described as a mechanism for bacterial communication in *Pseudomonas aeruginosa* and has since been found to be well conserved among plant-associated bacteria and secretes effector molecules directly into neighboring cells, similar to how a bacteriophage injects its genome into a host cell (Hood et al., 2010; Bernal et al., 2018). Some effector molecules have been identified as toxin-immunity protein pairs. The toxin is injected into a bacterial cell to kill it and thus decrease competition for essential nutrients and minerals, while the immunity protein stays in the donor cell to protect it from any toxin that may have not been secreted (Russell et al., 2011). Effector molecules can be hard to identify experimentally as they are often not present in high enough concentrations be to detected (Basler, 2015). However, bioinformatic research has shown that effector molecules are often in the same gene clusters or operons as the core genes (Basler, 2015). *Erwinia persicina* SR15 harbors two T6SS gene clusters that contain the core structural genes as well as hypothetical proteins of unknown function. The other strains isolated in this study, *E. persicina* SR13, 14, and 16, contain these T6SS genes as well, but the genes are not clustered as nice due to these genomes not being closed yet. Future work would benefit from investigating these unknown proteins to determine if they may, in fact, be effectors molecules for *E. persicina* that have not been discovered yet.

#### Phenolic acid decarboxylase

The discovery of a putative phenolic acid decarboxylase in the *E. persicina* genomes was surprising. Phenolic acid decarboxylases are enzymes that degrade phenolic acids and have been characterized in *Bacillus* and *Lactobacillus* species in the context of bovine ruminal digestion of plant material and malolactic fermentation of wine (Zago et al., 1995; Cavin et al., 1997; Tran et al., 2008). Plants produce phenolic acid compounds as both antimicrobials and signaling molecules (Li et al., 2009; Bhattacharya et al., 2010; Mandal et al., 2010; Joshi et al., 2016). The putative decarboxylase is highly conserved in all *E. persicina* strains (99–100% amino acid identity), but is absent from the other *Erwinia* and *Pectobacterium* strains examined in this study (Supplementary Table S1). While the phenolic acid decarboxylase is absent from *P. carotovorum* WPP14, it is found in other *Pectobacterium* species, including *P. carotovorum* SCC1 and *P. atrosepticum* SCRI1043 (data not shown). The putative phenolic acid decarboxylase in *E. persicina* is similar to the functionally characterized phenolic acid decarboxylase found in *Bacillus subtilis* (53% amino acid identity) and *Lactobacillus plantarum* (52% amino acid identity). It is possible that this enzyme is an adaptation to tolerate antimicrobial compounds produced by the host or to utilize another nutrient source that is provided by the plant host. Future research is needed to further characterize the phenolic acid decarboxylase found in *E. persicina* to confirm its function and investigate its significance in colonizing plant hosts and if it contributes to disease. Which phenolic acids are degraded by the decarboxylase, and at which point in the disease process the decarboxylase is utilized by the pathogen are important aspects to understanding how the pathogen resides in the plant environment and causes disease.

#### Iron uptake genes

Iron is needed for many biological functions, so it is a main source of competition between bacterial strains as well as in plant-pathogen interactions (Franza et al., 2004; Expert et al., 2012; Born et al., 2016). Siderophores work as chelators to find iron in the environment and bring it back to the bacterial cell it came from. Iron uptake proteins described as important for E. amylovora (Smits et al., 2010) are well conserved among all the organisms compared, with two exceptions: a putative copper receptor protein (OprC), involved in the adsorption of iron-containing siderophores, and a desferrioxamine siderophore synthesis protein (DfoC) which are missing from E. persicina strains (Table 6). Desferrioxamine siderophores are considered some of the strongest siderophores discovered so far, based on their ability to bind iron, and the genes responsible for the synthesis of one of those siderophores (EAMY_3238–3240) are only present in E. amylovora, pyrifoliae, and tasmaniensis (Smits and Duffy, 2011). Utilizing this siderophore may provide these organisms with an advantage when colonizing the plant environment. The pink pigmentation observed during soft rot infection by *E. persicina* is caused by the iron held by a siderophore called proferrorosamine, and the genes responsible for the siderophore, *rosA-G,* were initially characterized in *E. rhapontici* P45 (*E. rhapontici*) (Born et al., 2016). The whole cluster of *ros* genes is present in all *E. persicina* and *E. rhapontici* strains in this study, and absent from the other *Erwinia* species and the common soft rot agent (Table 6). The gene, *rosF*, responsible for a polyketide synthase has some similarity to polyketide synthases in *E. amylovora* (44% amino acid identity) and *P. carotovorum* (37% amino acid identity; Table 6). This siderophore has an inhibitory effect on *E. amylovora* in co-cultures with *E. rhapontici* when compared to co-cultures with deficient mutants (Born et al., 2016). It is unclear if this advantage extends to *E. persicina* as well, but if it does, this would provide a mechanism for how *E. persicina* is able to persist in the plant environment.

**Table 6:** Iron transport and pink pigmentation production in *Erwinia persicina* strains.

Gene type	Gene	Locus tag^a^	Ea^b^	Ep B64	Ep 102418	Ep 13/14	Ep 15/16	Er	Et	Pc
			Amino acid identity (%)							
Iron transport	Iron transport	EAMY_1080	100	84	84	84	84	84	90	24
	Ferric uptake regulator *fur*	EAMY_1148	100	97	97	97	97	97	97	93
	Iron transport	EAMY_1761	100	70	71	70	71	71	78	56
	Fe/Cu transport	EAMY_1821	100						84	26
	Siderophore biosynthesis	EAMY_3238	100	45	45	45	45	44	89	44
	Siderophore biosynthesis	EAMY_3240	100						91	
	Iron transport *foxR*	EAMY_3241	100	38	38	38	38	70	91	42
Siderophore synthesis (pink pigmentation)	*rosA*	AMB18979		79	79	79	79	79		
	*rosB*	AMB18978		72	72	72	72	74		
	*rosC*	AMB18977		91	91	91	91	91		
	*rosD*	AMB18976		87	87	87	87	85		
	*rosE*	AMB18975		88	88	88	88	90		
	*rosF*	AMB18974	44	82	83	83	82	85		37
	*rosG*	AMB18973		85	85	85	85	89		

a EAMY locus tags are from *Erwinia amylovora* CFBP1430; AMB locus tags are from *Erwinia rhapontici* P45.

b Organisms are as follows: Ea, *Erwinia amylovora* CFB1430; Ep B64, *Erwinia persicina* B64; Ep 102418, *Erwinia persicina* NBRC 102418; Ep 13/14, *Erwinia persicina* SR13/14; Ep 15/16, *Erwinia persicina* SR15/16; Er, *Erwinia rhapontici* MAFF 311153; Et, *Erwinia tasmaniensis* Et1/99; and Pc, *Pectobacterium carotovorum* WPP14.

#### Quorum sensing genes

Quorum sensing genes which are important regulators in causing disease (Von Bodman et al., 2003) were surveyed. Quorum sensing works through the use of a density-dependent transcriptional regulator, LuxR or ExpR, that is only activated when a certain amount of the signal, synthesized by LuxS or ExpI, is around indicating the presence of enough bacteria to produce disease symptoms before being recognized by the host (Crépin et al., 2012). Both sets of quorum-sensing genes used in the comparisons, *luxR/S* and *expR/S*, encode transcriptional regulators and signal synthases, and are well conserved in all the organisms examined (Supplementary Table S1). Soft rot bacteria use quorum sensing to regulate the production of pectolytic enzymes until the circumstances are right to cause disease (Põllumaa et al., 2012). Other plant pathogens use them to regulate various virulence factors such as biofilm formation, antibiotic production, and motility (Von Bodman et al., 2003). Based on that information it is expected that the plant bacteria in this study would all have quorum sensing capabilities, and they do. All strains examined in this study, including the *E. persicina* strains, encode genes for proteins that are similar to LuxR/S (36–100% amino acid identity) or ExpR/I (28–100% amino acid identity). The ability to control gene expression by quorum sensing is critical to causing soft rot and other plant diseases for the organisms investigated here.

#### Amylovoran biosynthesis

The production of amylovoran is an important virulence factor for *E. amylovora* when causing fire blight of apples and pears. Mutants deficient in the genes responsible for the synthesis of the exopolysaccharide, *amsD/E*, have decreased virulence on pears than the wildtype (Steinberger and Beer, 1988). Prior to being described as a soft rot agent, *E. persicina* was also found to be an opportunistic pathogen of legumes and alfalfa causing symptoms similar to fire blight, although less severe (González et al., 2005, 2007; Zhang and Nan, 2012, 2014). Genes similar to *amsD* from *E. amylovora* are present, at low identities (24–33% amino acid identity), in all strains of *E. persicina* in this study (Supplementary Table S1). The presence of the similar *amsD* gene in *E. persicina* could contribute to its ability to cause necrotic leaf spots. Amylovoran contributes to the ability of *E. amylovora* to move throughout an infected plant host and produce biofilms that occlude leaf tissues resulting in death of plant cells. It is likely that a similar amylovoran gene in *E. persicina* has a similar function. More research would need to be done before any definitive statements could be made.

#### *Erwinia persicina* plasmids are a potential adaption to the plant environment

**Figure 5:**
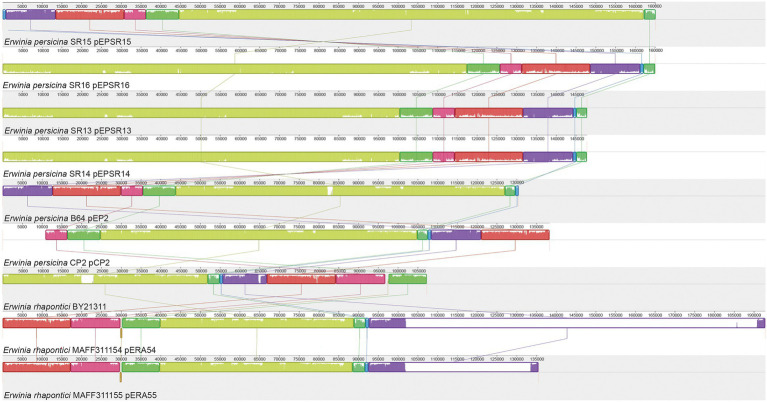
MAUVE alignment of *Erwinia* plasmids. Alignment of *E. persicina* and *E. rhapontici* plasmids. Plasmid sequences from *E. persicina SR13, SR14,* SR15, SR16, *E. persicina* B64 plasmid pEP2, *E. persicina* CP2, and *E. rhapontici* BY21311, MAFF311154 and MAFF311155 were aligned using the MAUVE software (Darling et al., 2010). Identically colored blocks denote homologous regions. Bar heights within blocks of homologous regions correlate with the level of conservation shared between the plasmid sequences. The numerical scale is in base pairs.

*Erwinia persicina* SR13 and 14 contain identical plasmids, each with a size of 148 kbp and 54.6% GC content. *E. persicina* SR15 and 16 also have identical plasmids, each 165 kbp in size and 54.3% GC content. The plasmids in SR13/14 and SR15/16 were 99.3% identical with 89% overlap (Figure 5). The plasmid in SR15/16 was 99% identical (with 78% overlap) to pEP2 from *E. persicina* B64 (Cho et al., 2019). In addition, high similarity was found between a number of large plasmids in other *E. persicina* and *E. rhapontici* strains (Figure 5). There are interesting genes present in the *E. persicina* SR13/14/15/16 plasmids that have the potential to be important for adaptation to the plant environment. Plants release reactive oxygen species during the immune response. Two genes were similar to a putative catalase gene and a peptide methionine sulfoxide reductase (*mrsA*) that have been demonstrated in *Escherichia coli* as important for repairing oxidative damage (Moskovitz et al., 1995). Similar genes were found in the *E. amylovora* pEA29 plasmid and the other *E. persicina* and *E. rhapontici* plasmids, these proteins could help mend proteins damaged by the release of reactive oxygen species by the plant host (McGhee and Jones, 2000).

There were three putative fimbriae loci and a H-NS (histone-like nucleoid structuring) DNA-binding protein, a negative regulator of fimbriae (Korea et al., 2010). Fimbriae are important to adhering to surfaces and cells (McGhee and Jones, 2000) and can potentially contribute to colonization of plant hosts by plant pathogens (Korea et al., 2010).

Other proteins were colicin V secretion proteins (*cavAB*) without the gene that codes for colicin itself (*cavC*) and the entire set is absent on the SR13/14 plasmid. Colicins are bacteriocins often used in competition with other bacteria (Gilson et al., 1990). Thus, the presence of the putative colicin export genes could provide the strains harboring those plasmids with a competitive advantage over other bacteria in the plant environment. However, it should be noted that a putative colicin gene has not been identified yet.

There were also genes responsible for thiamine metabolism (*thioFGSO*) present on the *E. persicina* and *E. rhapontici* plasmids. Genes responsible for thiamine production were also present on the *E. amylovora* pEA29 plasmid. Thiamine is an essential vitamin, so the ability to synthesize it themselves would be beneficial to any bacteria, and plasmid-cured strains of *E. amylovora* have lower virulence than the wildtype (McGhee and Jones, 2000).

Lastly, there was an entire pathway for the degradation of aromatic amino acids or 4-hydroxyphenylacetic acid, which is also present on the SR13/14 and pEP2 plasmids. This degradation pathway consists of 11 genes (*hpaCBAIHFDEGR*), their proteins show between 59 and 88% amino acid with the *E. coli* homologs and this pathway likely provides another potential adaptation to utilize aromatic amino acids, phenolic acids, similarly to the phenolic acid decarboxylase mentioned earlier (Prieto et al., 1996). This may provide *E. persicina* strains harboring those pathways with additional sources for nutrients and provide an additional advantage in the plant environment. The functions of these large *Erwinia* plasmids will need to be verified in future work where the *E. persicina* strains are cured of their plasmids and are evaluated based on their ability to or severity of causing soft rot and their ability to utilize phenolic acids as nutrient sources.

## Conclusion

In conclusion, isolates SR13-16 harbor genes known to be responsible for causing soft rot, such as pectolytic enzymes and quorum sensing proteins, and surviving the plant environment, like iron uptake proteins. The presence of a phenolic acid decarboxylase that is absent for other *Erwinia* plant pathogens but is present in other soft rot agents is a potential adaption for the plant environment for soft rot pathogens. One question that still remains unanswered is the secretion system involved in releasing the pectolytic enzymes responsible for causing soft rot. The absence of the Out cluster in the Type II secretion system must mean that another, not yet identified, system is able to secrete the enzymes while causing disease. It is also possible that another secretion system, that has already been characterized with other functions, is able to compensate for the lack of the Out cluster. Future work is needed to address both the phenolic acid decarboxylase, which could be the full characterization of the protein or investigating the implications of the protein when colonizing the plant, and identifying the secretion system that is compensating for the lack of the Out cluster. Together, this study provides the first genomic analysis of a recently described soft rot agent, *E. persicina*, and adds to the growing body of knowledge about the devastating soft rot disease through comparisons of key virulence factors with another soft rot agent and other *Erwinia* species.

## Data availability statement

The datasets presented in this study can be found in online repositories. The names of the repository/repositories and accession number(s) can be found at: https://www.ncbi.nlm.nih.gov/genbank/, samn29758759 https://www.ncbi.nlm.nih.gov/genbank/, samn29758760 https://www.ncbi.nlm.nih.gov/genbank/, samn29758761 https://www.ncbi.nlm.nih.gov/genbank/, samn29758762.

## Author contributions

NT, CW, and SS-E designed the project and analyzed the data. CW, CE, EN, ML, FR-S, and KS carried out the experiments. CW wrote the manuscript with editorial help from SS-E and NP. All authors contributed to the article and approved the submitted version.

## Funding

This project was funded by the College of Agriculture and Life Sciences, Iowa State University.

## Conflict of interest

The authors declare that the research was conducted in the absence of any commercial or financial relationships that could be construed as a potential conflict of interest.

## Publisher’s note

All claims expressed in this article are solely those of the authors and do not necessarily represent those of their affiliated organizations, or those of the publisher, the editors and the reviewers. Any product that may be evaluated in this article, or claim that may be made by its manufacturer, is not guaranteed or endorsed by the publisher.
