# Supplementary material for: Genome analysis of Erwinia persicina reveals implications for soft rot pathogenicity in plants
Source: Front Microbiol. 2022 Oct 28;13:1001139. doi: 10.3389/fmicb.2022.1001139 (PMC9650351; doi:10.3389/fmicb.2022.1001139)
Supplement: Supplementary file 1 [file Table_1.docx]

**Table S1 Quorum sensing, exopolysaccharides, phenolic acid decarboxylase, and CRISPR systems in *Erwinia persicina***

| **Gene type** | **Gene** | **Locus tag^a^** | **Ea^b^** | **Ep B64** | **Ep 102418** | **Ep 13/14** | **Ep 15/16** | **Er** | **Et** | **Pc** |
| --- | --- | --- | --- | --- | --- | --- | --- | --- | --- | --- |
|  |  |  | Amino acid identity (%) | | | | | | | |
| Quorum sensing | Transcriptional regulator *luxR* | EAMY_0501 | 100 | 78 | 78 | 78 | 78 | 77 | 36 | 62 |
|  | AI-2 production *luxS* | EAMY_0812 | 100 | 89 | 89 | 89 | 89 | 89 | 98 | 85 |
|  | Transcriptional regulator *expR* | EAMY_1411 | 100 | 66 | 67 | 66 | 67 | 70 | 84 | 28 |
|  | AHL synthase *expI* | EAMY_1412 | 100 | 72 | 72 | 72 | 72 | 73 | 75 |  |
| Exopolysaccharides | Amylovoran biosynthesis *amsD* | EAMY_2247 | 100 | 33 | 33 | 33 | 33 | 33 | 31 |  |
|  | Amylovoran biosynthesis *amsE* | EAMY_2246 | 100 | 30 |  | 24 | 24 | 24 |  |  |
|  | Levan biosynthesis | EAMY_3695 | 100 |  |  |  |  |  | 91 |  |
| Phenolic acid decarboxylase | *padC* | WP_161975944 |  | 100 | 99 | 100 | 100 | 85 |  | ** |
| CRISPR system | CRISPR Cas2 | EAMY_2813 | 100 |  |  |  |  |  |  | 82 |
|  | CRISPR Cas3 | EAMY_2820 | 100 |  |  |  |  |  | 28 | 33 |
| ^a^EAMY locus tags are from *Erwinia amylovora* CFBP1430; WP_161975944 is from *Erwinia persicina* SR15  ^b^ Abbreviations of organisms are as follows: Ea=*Erwinia amylovora* CFB1430*,* Ep B64=*Erwinia persicina* B64, Ep NBRC102418=*Erwinia persicina* NBRC 102418, Ep13/14=*Erwinia persicina* SR13/14, Ep 15/16=*Erwinia persicina* SR15/16, Er*=Erwinia rhapontici* MAFF 311153, Et=*Erwinia tasmaniensis* Et1/99, and Pc=*Pectobacterium carotovorum* WPP14  **Is present with 74% amino acid identity in *Dickeya dadantii* 3937 | | | | | | | | | | |
